# Neurotrophic, Gene Regulation, and Cognitive Functions of Carboxypeptidase E-Neurotrophic Factor-α1 and Its Variants

**DOI:** 10.3389/fnins.2019.00243

**Published:** 2019-03-19

**Authors:** Lan Xiao, Xuyu Yang, Y. Peng Loh

**Affiliations:** Section on Cellular Neurobiology, Eunice Kennedy Shriver National Institute of Child Health and Human Development, National Institutes of Health, Bethesda, MD, United States

**Keywords:** neuroprotection, neurotrophic factor, hippocampus, neurodevelopment, FGF2, BCL2, WNT/beta-catenin, stress

## Abstract

Carboxypeptidase E, also known as neurotrophic factor-α1 (CPE-NFα1), was first discovered as an exopeptidase and is known to work by cleaving C-terminal basic amino acids from prohormone intermediates to produce mature peptide hormones and neuropeptides in the endocrine and central nervous systems, respectively. CPE-NFα1 also plays a critical role in prohormone sorting and secretory vesicle transportation. Recently, emerging studies have indicated that CPE-NFα1 exerts multiple non-enzymatic physiological roles in maintaining normal central nervous system function and in neurodevelopment. This includes potent neuroprotective and anti-depressant activities, as well as stem cell differentiation functions. In addition, N-terminal truncated variants of CPE-NFα1 have been identified to regulate expression of important neurodevelopmental genes. This mini-review summarizes recent advances in understanding the mechanisms underlying CPE-NFα1’s function in neuroprotection during stress and aspects of neurodevelopment.

## Introduction

CPE-NFα1, a member of the M14 metallocarboxypeptidase family was discovered in 1982 in bovine adrenal medulla and named as enkephalin convertase due to its enzymatic activity in processing enkephalin precursor into its mature form (Fricker and Snyder, 1983). Since then, CPE-NFα1 has been shown to cleave C-terminal basic amino acids from the intermediates generated by proprotein convertases’ action on prohormones, thereby producing bioactive hormones and neuropeptides (Hook et al., 1982; Fricker and Snyder, 1983; Fricker, 1988). In the central nervous system, CPE-NFα1 also functions as a regulated secretory pathway sorting receptor, secretory vesicle transport regulator and mediates synaptic vesicle localization to the active zone for release (Cawley et al., 2012; Ji et al., 2017). Recent studies have indicated that CPE-NFα1 is a new neurotrophic factor functioning extracellularly, independent of its enzymatic activity, in the adult and embryonic central nervous system (Ji et al., 2017). Human mutations of CPE-NFα1 have been associated with obesity, diabetes, infertility, learning disabilities, and Alzheimer’s disease (AD) (Alsters et al., 2015; Cheng et al., 2016a). This review presents recent advances concerning the molecular structure, distribution, and multifunctional roles of CPE-NFα1 and its variants in the central nervous system in health and disease.

## Molecular Structure and Biosynthesis of CPE-NFα1 and Its Variants

**FIGURE 1:**
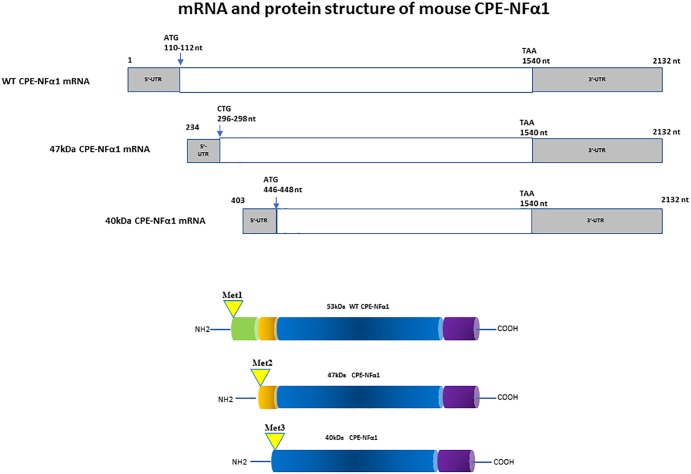
mRNA and protein structure of mouse CPE-NFα1. There are three CPE-NFα1 transcripts: a 2.3 kb CPE-NFα1 mRNA WT transcript and two variants, 1.9 and 1.73 kb transcripts generated by alternative transcription start sites in mouse embryonic brain. CPE-NFα1 mRNA WT transcript produces a 53 kD protein, while the two mouse CPE-NFα1 mRNA variants produce two N-terminal truncated CPE-NFα1 proteins with 47 and 40 kD, respectively. Numbers in the 47 and 40 kD CPE mRNA refer to their relative position in the wt-CPE mRNA.

In mammals, WT-CPE-NFα1 gene consists of nine exons and encodes a 476 amino acid polypeptide (Jung et al., 1991; Cawley et al., 2012). Two CPE-NFα1 mRNA transcripts, 2.4 and 1.7 kb in size, respectively, have been identified by Northern Blot analysis and DNA sequencing from human cancer cells. The 2.4 kb CPE-NFα1 transcript encodes a 53 kD WT-CPE/NFα1 and the 1.7 kb transcript encodes an N-terminal truncated 40 kD CPE-NFα1 due to the intra-exonic splicing in exon1 (Yang et al., 2017). Both transcripts have also been detected in Northern blot of human hippocampal mRNA extract (Yang, our unpublished data). Three CPE-NFα1 transcripts, a 2.3 kb WT transcript and two CPE-NFα1 mRNA variants, 1.9 and 1.73 kb in size (Figure 1) have been identified in mouse embryonic brain. Unlike in human cancer cells, the two mouse CPE-NFα1 mRNA variants are generated by using alternative transcription start sites, each of them encodes an N-terminal truncated CPE-NFα1 with a molecular weight of 47 and 40 kD, respectively (Yang et al., 2017; Xiao et al., 2018).

**FIGURE 2:**
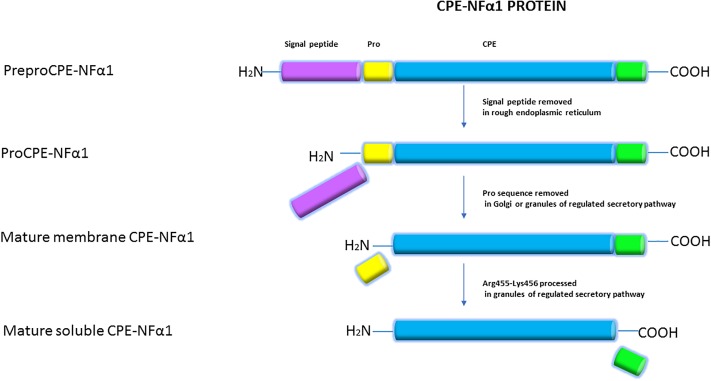
Synthesis of CPE-NFα1. CPE-NFα1 is synthesized as preproCPE-NFα1 which contains a 25-amino acid signal peptide. In the cisternae of rough endoplasmic reticulum, the signal peptide is removed. Then, the 17-amino acid pro peptide is cleaved in the Golgi complex or granules of the regulated secretory pathway. Further processing at Arg455-Lysine 456 produces a soluble form of CPE-NFα1, with the unprocessed form being membrane associated.

The CPE-NFα1 protein consists of a signal peptide, a catalytic domain, and a C-terminal domain. A 3-D structure model of CPE-NFα1 shows a zinc binding site in the enzymatic domain, a prohormone sorting signal binding site, an amphipathic α-helical transmembrane domain, and a cytoplasmic tail that interacts with microtubule proteins for vesicle transport (Cawley et al., 2012). Mature CPE-NF α1 has 476-amino acids. It is synthesized as preproCPE-NFα1 that has a 25-amino acid signal peptide located at the N-terminal that directs proCPE-NFα1 to the cisternae of the rough endoplasmic reticulum (ER) where it is removed (Figure 2). The proCPE-NFα1 is then transported to the granules of the regulated secretory pathway through the Golgi complex where 17-amino acids comprising the pro-region are removed (Song and Fricker, 1995). Further processing at Arg455-Lysine 456 (Fricker and Devi, 1993) in the secretory granules yields a soluble form of CPE-NFα1 (50 kDa), while the 53 kD unprocessed form is associated with the granule membrane, with some molecules assuming a transmembrane orientation (Cawley et al., 2012).

## Distribution of CPE-NFα1 and CPE-NFα1-ΔN

In mammals, CPE-NFα1 is generally distributed in the endocrine and nervous system, with the highest concentration in the brain (Strittmatter et al., 1984). Immunohistochemical studies showed CPE-NFα1 is located in neuropeptide-rich regions of the brain and endocrine system, for example, in the hypothalamus, pituitary, adrenal medulla, paraventricular nucleus, hippocampus, and amygdala (Hook et al., 1985; Lynch et al., 1990). Consistently, CPE-NFα1 mRNA is also found to be highly expressed in these brain regions (Birch et al., 1990; MacCumber et al., 1990; Zheng et al., 1994). The retina and olfactory bulb are also highly enriched in CPE-NFα1 (Zhu et al., 2005). In the retina, CPE-NFα1 is localized to the photoreceptors and is involved in synaptic transmission to the inner retina (Zhu et al., 2005). At the cellular level, CPE-NFα1 is mainly distributed in the trans-Golgi network and dense core secretory granules of endocrine cells and peptidergic neurons (Cawley et al., 2012). CPE-NFα1 exists as two active forms, the soluble and membrane form (Figure 2). The soluble form of CPE-NFα1 functions as an enzyme that cleaves C-terminal basic residues such as arginine and lysine from intermediates derived from neuropeptide precursors or prohormones to produce biologically active peptides or hormones. The membrane form of CPE-NFα1 acts as a sorting receptor at the trans-Golgi network to direct prohormones to the regulated secretory pathway granules, and mediates secretory granule movement (Cawley et al., 2012). In contrast, the N-terminal truncated (CPE-NFα1-ΔN) forms which lack a signal peptide do not enter the secretory pathway. The 40 kD form of CPE-NFα1-ΔN has been shown to be localized in the cytoplasm and to be able to translocate into the nucleus where it exerts its effects on gene regulation (Qin et al., 2014) (see summary in Table 1).

**Table 1:** Comparison of CPE-NFα1 WT and CPE-NFα1-ΔN.

	CPE-NFα1 WT	CPE-NFα1-ΔN
Biosynthesis	First transcription start site	Alternative transcription start sites
Intracellular location	Regulated secretory vesicles	Cytoplasm and nucleus
Distribution	Endocrine and CNS. Embryonic and adult brain	Embryonic, not adult brain in mouse
Soluble and membrane forms	Soluble and membrane associated	Soluble
Extracellular secretion	Secreted from vesicles to extracellular space	Not secreted
Signal peptide	Yes	No
Structure	–	Lacking N-terminal
Enzymatic activity	Yes	Weak enzymatic activity
Intracellular sorting receptor to regulatory secreted pathway	Yes	No
Facilitates vesicle trafficking via cytoplasmic tail	Yes	No

## Mice and Humans With CPE-NFα1 Mutation Exhibit Neurological Deficits

CPE-NFα1 knockout (KO) mice, in addition to exhibiting endocrinological deficits such as obesity, diabetes, and infertility due to lack of enzyme activity, display a variety of behavioral abnormalities as evidenced by deficits in learning and memory in Morris water maze, object preference, and social transmission of food preference tests (Woronowicz et al., 2008). They also display depressive-like behavior (Cheng et al., 2015, 2016a). CPE^fat/fat^ mice which have a Ser202Pro mutation and lack CPE-NFα1 have a similar phenotype to the knockout mouse and exhibit anxiety-like and depression-like behaviors (Rodriguiz et al., 2013). Interestingly, a human with a null mutation also demonstrated similar symptoms, such as obesity, diabetes, hypogonadotropic hypogonadism, and impaired intellectual ability (Alsters et al., 2015). In another study, a human mutation of CPE-NFα1 with three adenosines insertions was identified, this mutation was named QQ-CPE-NFα1. Mice bearing this mutation showed neurodegeneration in the hippocampus and prefrontal cortex, deficits in neurogenesis at the dentate gyrus and hyperphosphorylation of tau (Cheng et al., 2016a). Another human CPE-NFα1 mutation, T980C, which introduces a W235R change in the catalytic domain of CPE-NFα1 caused a loss of enzyme activity, neurotoxic accumulation in the ER, resulting in ER stress and cell death when overexpressed in Neuro 2A cells. This novel single nucleotide polymorphism in the CPE-NFα1 gene found in 12.5% of the AGI_ASP population may confer neurological disorders in humans (Cong et al., 2017). These observations suggest that CPE-NFα1 plays an important role in neurological function, and mutation of this gene can lead to cognitive and neurodegenerative disorders.

## CPE-NFα1 in Neurodegenerative Disorders and Stress

In the cerebral cortex of AD patients, an abnormal accumulation of CPE-NF α1 was detected in dystrophic neurites surrounding amyloid plaques (Pla et al., 2013). In an AD animal model of APPswe/PS1dE9 mice, similar pattern of changes were also found. Amyloid plaques were surrounded by aberrant accumulation of CPE-NFα1 and Secretogranin III (SgIII) (Pla et al., 2013). Peptidase activity analysis of the postmortem brain from AD patients showed that soluble enzymatic activity of CPE-NFα1 was significantly increased in the Brodmann Area 21 compared with control (Weber et al., 1992). In a Cathepsins B and L double knockout mouse model that demonstrated early-onset neurodegeneration and reduction in brain size, the expression of CPE-NFα1 was upregulated by 10-fold (Stahl et al., 2007). In experimental autoimmune encephalomyelitis (EAE) animal model, CPE-NFα1 has been mapped as a EAE-linked trait loci which is associated to the severity of the disease and shown to be downregulated (Ibrahim et al., 2001; Mazon Pelaez et al., 2005). These observations suggest that CPE-NFα1 is associated to the pathophysiology of neurodegenerative diseases.

Studies have shown that CPE-NFα1 expression is up-regulated after different types of stress. CPE-NFα1 mRNA and protein were both increased in the rat hippocampal CA1, CA3, and cortex regions 15 min after transient global ischemia followed by 8 h reperfusion (Jin et al., 2001). An accumulation of CPE-NFα1 precursor was found in mouse cortex after focal cerebral ischemia *in vivo* and in ischemic cortical neurons *in vitro*. Vice versa, mice lacking CPE-NFα1 were more vulnerable to focal cerebral ischemia (Zhou et al., 2004). Stress such as cat odor upregulated CPE-NFα1 gene expression in rat amygdala (Koks et al., 2004). Mild chronic restraint stress for 7 days up-regulated CPE-NF α1 expression in mouse hippocampal CA3 region (Murthy et al., 2013). Reduced levels of CPE-NFα1 were found in the offspring of pregnant ewes that experienced aversive interaction with human handling, along with abnormal corticolimbic dendritic spine morphology (Coulon et al., 2013). Primary cultured hippocampal neurons derived from CPE-NFα1-KO mice tend to die more rapidly than that from wild type (WT) mice (Cheng et al., 2013). Low-potassium-induced apoptosis in cultured primary cerebellar granule neurons from CPE-NFα1^+/−^ mice are much higher than that from CPE-NFα1^+/+^ mice (Koshimizu et al., 2009). Overexpression of CPE-NFα1 in rat primary hippocampal neurons prevented hydrogen peroxide-induced neurotoxicity (Woronowicz et al., 2008). All these studies indicate that stress modulates CPE-NFα1 expression in the central nervous system (CNS).

## CPE-NFα1 as a Neurotrophic Factor in Neuroprotection and Anti-Depression

**FIGURE 3:**
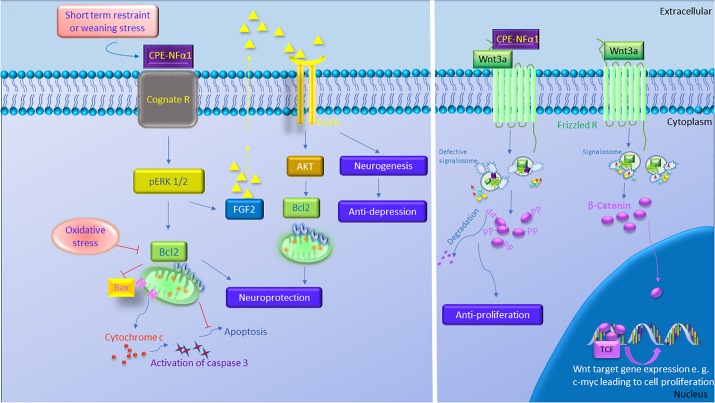
The multiple functions of CPE-NFα1 in the central nervous system (CNS). CPE-NFα1 plays multiple functions in the CNS independent of its enzymatic activity. Short term restraint or weaning stress increases CPE-NFα1 which binds to a cognate receptor and upregulates ERK1/2 and Bcl2, and decreases Bax and cytochrome c-induced activation of caspase 3 and apoptosis. CPE-NFα1 also increases FGF2 that activates AKT and Bcl2 signaling and increases hippocampal neurogenesis and produces antidepressive-like effect. CPE-NFα1 can also bind to Frizzled receptor – Wnt3a complex, enters the endosome that contains Wnt signaling factors and subsequently impairs the formation of signalosome. This results in β-catenin being degraded, rather than translocation into the nucleus and binds to T-cell factor transcription factor (TCF) to activate expression of Wnt target genes. 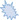
Signalosome 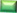
Wnt 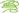
Fz 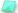
APC 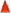
GSK-3β 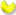
Axin.

CPE-NFα1 plays multiple intracellular roles in the CNS independent of its enzymatic activity. It functions as a regulated secretory pathway sorting receptor for proneuropeptides (Cool et al., 1997) and proBDNF (Lou et al., 2005), mediates anterograde transport of neuropeptide and BDNF vesicles to the plasma membrane via cytoplasmic tail interaction with dynactin/dynein (Park et al., 2008), and localizes synaptic vesicles to the actin-rich pre-active zone in hypothalamic neurons through actin binding protein γ-adducin (Lou et al., 2010). While the mechanisms underlying these cell biological actions of CPE-NFα1 are well understood, those governing the neuroprotective effects of CPE-NFα1 during stress are just emerging. In 2013, a study showed that CPE-NFα1 secreted from hippocampal neurons, acts as a neuroprotective factor extracellularly, independent of its enzymatic activity (Cheng et al., 2013). Secreted rat WT CPE-NFα1 and CPE-NFα1-E300Q (mutant form with no enzymatic activity) (Qian et al., 1999) in medium from transduced cultured primary rat hippocampal neurons protected them from cell death induced by H_2_O_2_-mediated oxidative stress and glutamate excitotoxicity. Results from experiments with recombinant WT CPE-NFα1 or CPE-NFα1-E342Q (mouse homolog of E300Q) demonstrated similar effects. In addition, hippocampal neurons from CPE-NFα1-KO mice at embryonic day 17 (E17) in culture exhibited higher death rate than WT E17 neurons, and this was reversed by treatment with recombinant WT CPE-NFα1 (Cheng et al., 2013). Studies on the mechanism underlying the neuroprotective action of CPE-NFα1 using rat primary hippocampal neurons in culture revealed that when these cells were stressed with H_2_O_2_, it activated the ERK and AKT signaling pathways, presumably by binding to a cognate receptor, which then up-regulated expression of Bcl2, a pro-survival mitochondrial protein, and down-regulated expression of Caspase 3 to mediate neuroprotection (Cheng et al., 2013) (see Figure 3).

**FIGURE 4:**
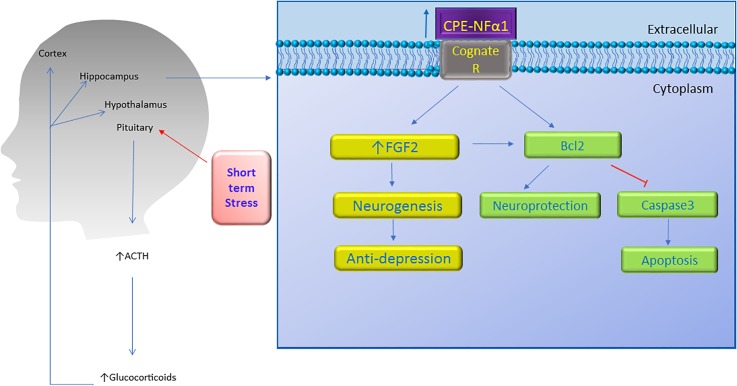
Neuroprotective effect of CPE-NFα1. Short term stress activates the hypothalamic-pituitary-adrenal axis which then increases ACTH and glucocorticoid secretion. The glucocorticoids upregulate CPE-NFα1 expression via glucocorticoid regulatory elements (GRE) on the promoter. CPE-NFα1 is secreted and possibly activates its cognate receptor which then produces antidepressant-like effects via increasing FGF2 expression and neurogenesis in the hippocampus. Additionally, CPE-NFα1 protects against neurodegeneration through upregulating Bcl2, a mitochondrial pro-survival protein, by inhibiting caspase 3-mediated apoptosis.

**FIGURE 5:**
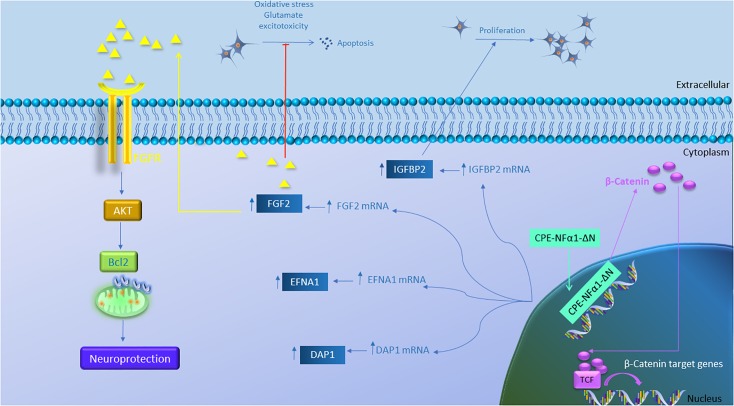
Gene regulation function of CPE-NFα1-ΔN in mouse embryonic neurons. CPE-NFα1-ΔN increases expression of FGF2, IGFBP2, EFNA1, and DAP1 in mouse embryonic cortical and hippocampal neurons. IGFBP2 promotes neuronal proliferation while FGF2 inhibits oxidative stress or glutamate-induced excitotoxicity. FGF2 binds its receptor and induces neuroprotective effects via activating the AKT and Bcl2 signaling cascade. CPE-NFα1-ΔN expressed in hippocampal neurons translocates into the nucleus and upregulates β-catenin expression. β-catenin then enters the nucleus and binds to T-cell factor (TCF) transcription factor to promote expression of Wnt target genes.

In an *in vivo* mouse model, complete degeneration of hippocampal CA3 region was observed in CPE-NFα1-KO mice at 4-weeks of age after weaning stress which included maternal separation, ear tagging, and tail snipping at 3 weeks of age (Woronowicz et al., 2008). CPE-NFα1-KO mice that were not subjected to the weaning process at 3 weeks of age showed no degeneration of the CA3 region examined at week 4, indicating that this neurodegeneration was not due to a neurodevelopmental defect. Treatment with oral carbamazepine at 50 mg/kg daily for 2 weeks beginning at 2 weeks of age with the weaning process at 3 weeks of age, revealed no degeneration of the CA3 region when examined at 4 weeks of age (Woronowicz et al., 2012). These observations suggested that the CA3 pyramidal neurons underwent apoptosis due to glutamate excitotoxicity during the social and physical stress following the weaning protocol. In contrast, WT-mice, did not show any degeneration of the CA3 region after weaning stress. In another mouse model, adult CPE^fat/fat^ mice lacking CPE-NFα1, also showed degeneration of the CA3 region (Zhou, personal communication). These findings together with the *in vitro* studies (Cheng et al., 2013) support the hypothesis that CPE-NFα1 acts as a neurotrophic factor to protect the pyramidal neurons in the CA3 region in the hippocampus from stress-induced degeneration. Since BDNF is expressed in CPE-NFα1-KO mice (Xiao et al., 2017), yet they showed neurodegeneration after weaning, indicates that BDNF could not protect the CA3 neurons in lieu of CPE-NFα1. Further demonstration that CPE-NFα1 is a neuroprotective factor *in vivo* during stress came from studies showing that chronic restraint stress of mice for 1 h/day for 7 days resulted in an increase in CPE-NFα1 mRNA and protein expression in the hippocampus, with no evidence of neurodegeneration despite increased circulating corticosterone levels under this stress paradigm (Murthy et al., 2013). This up-regulation in expression of CPE-NFα1 concurrs with *in vitro* evidence showing that the CPE-NFα1 promoter has a glucocorticoid binding domain and that dexamethasone up-regulated the expression of CPE-NFα1 (Murthy et al., 2013). These mice also showed an increase in phosphorylation of Akt and Bcl2 expression; however, Bax, a pro-apoptotic mitochondria protein was decreased in the hippocampus (Murthy et al., 2013). In contrast, CPE-NFα1-KO mice subjected to the same stress paradigm showed no change in Akt phosphorylation, a decrease in expression of Bcl2 protein and an increase in Bax protein in the hippocampus compared to WT mice (Murthy et al., 2013). *In vitro* and *in vivo* evidence taken together indicate that during emotional and physical stress, secretion of glucocorticoid increases CPE-NFα1 expression at the transcriptional and translational level in the hippocampus which in turn leads to neuroprotection of the CA1-3 neurons by acting extracellularly as a trophic factor to activate Erk or Akt signaling and increase Bcl2 pro-survival protein expression (see Figure 3, 4). CPE-NFα1 also up-regulates the expression of FGF2 (Cheng et al., 2015), which has been shown to mediate protection against amyloid beta- or glutamate-induced neurotoxicity in hippocampal or cortical neurons via the Akt-Bcl2 signaling pathway (Qin et al., 2014; Cheng et al., 2016b; Figure 3, 5).

Behavioral analysis revealed that the CPE-NFα1-KO and the Cpe^fat/fat^ mice exhibited depressive-like behavior as evidenced by increased immobility time in the forced-swimming test (Rodriguiz et al., 2013; Cheng et al., 2015). CPE-NFα1-KO mice showed decreased levels of FGF2 (Cheng et al., 2015), a protein demonstrated to be decreased in post mortem brains of patients with major depressive disorder (Evans et al., 2004). In addition, after long-term chronic restraint stress, mice showed depressive-like behavior, and reduced CPE-NFα1, FGF2 and doublecortin, a marker for neuroblasts in their hippocampus. Interestingly, injection of 5 ng/g recombinant FGF2 into CPE-NFα1-KO mouse for 30 consecutive days completely reversed the decreased number of doublecortin positive neurons in the subgranular zone of hippocampus and depressive-like behaviors as evidenced by decreased immobility time in the forced-swim test (Cheng et al., 2015). *In vitro* studies showed that incubation of rat cultured hippocampal neurons with CPE-NFα1 enhanced FGF2 mRNA and protein expression which was inhibited by the Sp1 inhibitor mithramycin A, a transcription inhibitor actinomycin, and ERK inhibitor U0126, suggesting that CPE-NFα1 can upregulate FGF2 via ERK-Sp1 signaling cascade (Cheng et al., 2015). Thus, CPE-NFα1 may act as an anti-depressant through up-regulating FGF2 expression in the hippocampus.

## Role of CPE-NFα1 and CPE-NFα1-ΔN Variants in Neurodevelopment

In mice and rats, CPE-NFα1 mRNA is expressed during embryogenesis as early as E5.5 mainly in the developing nervous system (Zheng et al., 1994; Selvaraj et al., 2017). In mice, the expression of CPE-NFα1-WT and the two CPE-NFα1-ΔN transcripts were detected in E8.5 embryonic brain and their expression peaked at embryonic day (E)10.5, then decreased from E12.5 to E16.5, and increased again at postnatal day 1 (P1). Interestingly, none of the CPE-NFα1-ΔN mRNAs were found in adult mouse hippocampus or other organs such as liver, heart or lung, suggesting their critical role during embryonic neurodevelopment (Xiao et al., 2018). Studies of dendritic arborization in pyramidal layer V of cerebral cortex and hippocampal CA1 region in 14-week-old CPE-NFα1-KO mice revealed abnormal dendritic pruning and increased number of non-functional D-type spines (Woronowicz et al., 2010), suggesting a role of CPE-NFα1 in modeling the cytoarchitecture of the brain during development. Indeed, CPE-NFα1 has been shown to regulate NGF-induced neurite outgrowth via interacting with Wnt-3a and Wnt-5a in PC12 cells and mouse primary cortical neurons (Selvaraj et al., 2015). CPE-NFα1 is highly expressed in neural stem cells (Lee et al., 2012) and has been shown to inhibit embryonic neural stem cell proliferation by downregulating Wnt signaling pathway and β-catenin (Selvaraj et al., 2017). Studies have revealed that CPE-NFα1 forms a complex with Wnt3a ligand and Frizzled receptor to inhibit the Wnt3a signaling cascade (Figure 3; Skalka et al., 2013). Moreover, exogenous application of CPE-NFα1 and a non-enzymatic form of CPE-NF α1(E300Q) induced differentiation of E14.5 cortical neural stem cells into astrocytes via activating the ERK1/2-Sox9 signaling pathway to enhance glial fibrillary acidic protein (GFAP) expression (Selvaraj et al., 2017). This corroborates the 49% decrease in astrocyte numbers observed in the neocortex of mouse neonates *in vivo* at postnatal day 1 (P1). In another study, electroporation of CPE-NFα1 shRNA into E14.5 mouse embryos significantly decreased migration of neurons to cortex plate at E17.5, possibly due to the failure of transition from multipolar neurons into a bipolar morphology in the intermediate zone (Liang et al., 2018). Neuronal migration seems to depend on the function of the CPE-NFα1 cytoplasmic tail which can interact with dynactin, an adaptor protein that interacts with microtubules. Furthermore, this study also showed that CPE-NFα1 regulates dendrite morphology *in vivo* and in rat primary cultured hippocampal neurons (Liang et al., 2018).

While CPE-NFα1 acts extracellularly as an embryonic stem cell differentiation and anti-proliferation factor, studies have indicated that CPE-NFα1-ΔN could serve as a master regulator of genes involved in neurodevelopment (Figure 5 and Table 2). CPE-NFα1-ΔN (40 kD variant), but not CPE-NFα1 (Xiao, our unpublished data) was found to significantly up-regulate expression of four genes, fibroblast growth factor 2 (FGF2) (Qin et al., 2014), insulin-like growth factor binding protein2 (IGFBP2), death associated protein (DAP1), and EphrinA1 in HT22, a hippocampal cell line and in primary mouse cortical neurons (Xiao et al., 2018). These three genes are known to mediate neuronal proliferation, cell death and neuronal migration, respectively (Murphy et al., 1990; Deiss et al., 1995; Feinstein et al., 1995; Levy-Strumpf and Kimchi, 1998; Martinez et al., 2004; Martinez and Soriano, 2005; Russo et al., 2005; Zechel et al., 2010). Overexpressing of CPE-NF α1-ΔN in HT22 cells promoted proliferation, but was inhibited by IGFBP2 siRNA, suggesting CPE-NFα1-ΔN regulates embryonic neuronal proliferation through increasing IGFBP2. In addition, CPE-NFα1-ΔN-induced FGF2 expression has a neuroprotective role against glutamate and H_2_O_2_ neurotoxicity in rat embryonic cortical neurons (Qin et al., 2014). Since FGF2 is also known to play a role in neuronal proliferation and neurogenesis in the developing mouse cortex, CPE-NFα1-ΔN may also regulate these events in the embryonic brain (Raballo et al., 2000). Moreover, CPE-NFα1-ΔN has been shown to up-regulate the expression of β-catenin in the Wnt pathway in HEK-293 cells (Skalka et al., 2013) and osteosarcoma cell lines, which promoted migration of these cells (Fan et al., 2018). We have shown that neuroprotection by CPE-NFα1-ΔN against glutamate excitotoxicity may also involve β-catenin and Wnt pathway. Glutamate treatment resulted in a decrease in β-catenin level and poor cell viability compared to untreated control embryonic rat cortical neurons. However, in CPE-NFα1-ΔN-transduced neurons treated with glutamate, this decrease in β-catenin did not occur and the cell viability was similar to control. Treatment of cortical neurons with XAV939 which stimulates degradation of β-catenin (Huang et al., 2009) showed that in the presence of XAV939, the neuroprotective effect of CPE-NFα1-ΔN against glutamate neurotoxicity was abolished. This suggests that the activation of the Wnt/β-catenin pathway may also contribute to the neuroprotective mechanism of CPE-NFα1-ΔN in embryonic neurons (Qin et al., our unpublished data). Additionally, β-catenin has been reported to mediate neuronal proliferation and differentiation of stem cells (Israsena et al., 2004). Thus, CPE-NFα1-ΔN may also regulate these processes during development via the Wnt signaling pathway.

**Table 2:** Neuroprotective and neurodevelopmental functions of CPE-NFα1 WT and CPE-NFα1-ΔN.

	Function	Reference
CPE-NFα1 WT	Regulates NGF-induced neurite outgrowth	Selvaraj et al., 2015
	Inhibits neuronal stem cell proliferation	Selvaraj et al., 2017
	Induces differentiation of neural stem cells into astrocytes	Selvaraj et al., 2017
	Regulates cortical neuron migration and dendrite arborization	Liang et al., 2018
	Protects primary cultured rat hippocampal neurons from H_2_O_2_-induced oxidative stress	Cheng et al., 2013
	CA3 completely degenerated after weaning, ear tag and tail clipping at 3-week of age and prevented by carbamazepine	Woronowicz et al., 2008, 2012
	Upregulates FGF2 and produces antidepressant-like effects	Cheng et al., 2015
CPE-NFα1-ΔN (40 kD)	Protects primary rat embryonic cortical neurons from glutamate and H_2_O_2_-induced apoptosis via FGF2	Qin et al., 2014
	Increases proliferation via upregulating IGFBP2	Xiao et al., 2018

## Conclusion

CPE-NFα1 is a multifunction protein (Table 2). In addition to being a prohormone and proneuropeptide processing enzyme, it is a sorting receptor and vesicle transport mediator. Recent studies have shown that it also plays a fundamental role in neurodevelopment, neurodegeneration, and cognitive functions. Null mutation of CPE-NFα1 in animal models and a human both produced similar deficits, including obesity, diabetes, and compromised cognitive function and memory. In addition, abnormal accumulation in the ER of a human mutant of CPE-NFα1 from an AD patient was found which caused neurotoxicity, neurodegeneration, and cognitive impairment in an animal model. *In vitro* studies using primary neurons in culture indicate that CPE-NFα1 acts extracellularly as a trophic factor, independent of enzymatic activity to mediate neuroprotection via activating several pro-survival signaling cascades such as upregulating Bcl2 and FGF2 signaling. Studies to demonstrate the neurotrophic effect *in vivo* will be necessary using transgenic mouse models to further support this hypothesis. Additionally, identifying a membrane receptor for CPE-NFα1 will be required to substantiate a receptor-mediated mechanism for activating signaling pathways for neuroprotection and stem cell differentiation. Furthermore, CPE-NFα1 and its derived peptides can be developed as potential therapeutic agents to treat neurodegenerative diseases. The 40 kD CPE-NFα1-ΔN variant has been shown to activate genes involved in neurodevelopment (Table 2). Further studies will be required to determine the precise molecular mechanism underlying CPE-NFα1-ΔN’s role in activating such genes during embryonic development.

## Data Availability

The datasets generated for this study are available on request to the corresponding author.

## Author Contributions

All authors listed have made a substantial, direct and intellectual contribution to the work, and approved it for publication.

## Conflict of Interest Statement

The authors declare that the research was conducted in the absence of any commercial or financial relationships that could be construed as a potential conflict of interest.
